# Decreased expression of microRNA-17 in hippocampal tissues and blood from mice with depression up-regulates the expression of PAI-1 mRNA and protein

**DOI:** 10.1590/1414-431X20208826

**Published:** 2020-09-07

**Authors:** Min Lv, Jing Li, Xinxue Gao, Yurong Hao, Fengxia Zhao

**Affiliations:** 1 Department of Psychology, The Second Children and Women's Healthcare of Jinan City, Jinan, China; 2 Department of Gynecology, The Second Children and Women's Healthcare of Jinan City, Jinan, China; 3 Department of Psychiatry, Jining Psychiatry Hospital, Jining, China; 4 Cardiac Intensive Care Unit, Affiliated Hospital of Jining Medical University, Jining, China

**Keywords:** microRNA-17, PAI-1, Hippocampal tissues, Blood, Depression, Mouse model

## Abstract

This study determined the expression of plasminogen activator inhibitor-1 (PAI-1) and microRNA (miR)-17 in a mouse depression model. Forty male mice were divided evenly into control and depression groups. A chronic unpredictable mild stress (CUMS) model was constructed. qRT-PCR was used to determine the expression of PAI-1 mRNA and miR-17. Western blotting and ELISA were used to determine expression of PAI-1 protein. Dual luciferase reporter assay was carried out to identify direct interaction between miR-17 and PAI-1 mRNA. The mice with depression had elevated PAI-1 mRNA and protein in hippocampal tissues and blood. Expression of miR-17 was decreased in hippocampal tissues and blood from mice with depression. miR-17 bound with the 3′-UTR of PAI-1 mRNA to regulate its expression. This study demonstrated that miR-17 expression in hippocampal tissues and blood from mice with depression was decreased while expression of PAI-1 mRNA and protein was up-regulated. miR-17 participated in depression in mice by regulating PAI-1.

## Introduction

With the increase of competition pressure in modern society, the number of people suffering from depression is increasing day by day (1,2). Decreased hippocampal volume and nerve density are closely associated with depression (3). Animal experiments also confirm the disorder and looseness of the hippocampal neurons in animal models for depression (4). In addition, the nuclei of hippocampal neurons are crinkled, and the nuclear membrane becomes concave-convex (5).

Cerebrovascular lesions have always been an important topic in depression research. Plasminogen activator inhibitor-1 (PAI-1) is a kind of serine protease inhibitor that can inactivate plasminogen activators tissue-type plasminogen activator (tPA) and urokinase-type activator (uPA), inhibit fibrinolytic process, and cause hemorheological changes (6). The tPA/PAI-1-plasmin system widely exists in the central nervous system of human beings (7), and participates in a variety of molecular mechanisms in the brain (8). The expression level of PAI-1 in depressed women is higher than that in normal group (9). A single nucleotide polymorphism in the *PAI-1* gene is found to be associated with antidepressant response (10).

MicroRNAs (miRNA or miR) play an important role in regulating gene expression at the post-transcriptional level, and about 50% of protein-coding genes are regulated by miRNA (11). Expression of miRNA is found to be disordered in autopsy tissues of patients with depression, and further investigation shows that many downstream targets regulated by the miRNA are related to depression (12). miRNA can regulate central nervous system activities such as reward feedback, circadian rhythm, and cognitive performance (13-15), and abnormality of these activities is closely related with depression. miR-17 is a newly discovered miRNA molecule that plays an important regulatory role in many kinds of human diseases, and miR-17 92 gene clusters are also associated with human depression (16). However, there is no report on the regulation of PAI-1 by miR-17 in depression. In the present study, we tried to elucidate the mechanism by which miR-17 regulates PAI-1 expression in depression and provide a theoretical basis for the diagnosis, prevention, and treatment of the disease.

## Material and Methods

### Animals

A total of 40 male Kunming mice were obtained from Chongqing Tengxin Biotech Company (<http://www.cqtx123.com>, China) with a certificate numbered SCXK(Yu)2016-0028. The mice weighed 18-22 g. The Reduction, Replacement, and Refinement animal welfare principle was followed during the experiments. One week before experiments, the mice were maintained in cages (45×30×30 cm) with 12 h light/dark cycles. In the last 3 days, the mice received adaptive training of sugar water (one bottle of 2% sucrose water and one bottle of pure water; the positions of the two bottles were exchanged every 1 h). The mice were evenly and randomly divided into control group and depression group. Every five mice in the control group were raised in the same cage, while each mouse in the depression group was raised in a single cage.

To construct the chronic unpredictable mild stress (CUMS) model, the mice in the depression group were stimulated with one of the following stimulations each day for 35 consecutive days (17): diurnal upside down for 24 or 48 h, repeated cage tilt for 12 or 24 h (tilt direction was changed every 4 h), water deprivation for 6 or 12 h, fasting for 12 or 24 h, metal impact noise (2 h), damp (mild and severe) cushion for 12 or 24 h, foreign body, no cushion, suspension for 6 h, tail clipping, cage sharing, horizontal shaking, cage swapping, and change of feeding environment. From the fourth week on, body weight, amount of food and drink intake, percentage of sucrose water consumption, and open field activity were examined.

Mice that had significantly different body weight, percentage of sucrose water consumption, and open field activity after stimulations were considered to be a successful depression model. In the body weight test, the body weight and intake of food and water of each mouse were recorded one day before the beginning of the experiment and 28 and 35 days after. In the sucrose water consumption test, the percentage of sucrose water consumed by each mouse was recorded one day before the beginning of the experiment and 28 and 35 days after, according to a previously published report (18). Sugar water preference was calculated by sucrose water consumption/total drink consumption ×100%. In the open field test, a box with dimensions of 100×100×40 cm was used, and the field was divided into 25 squares with equal areas (19). Placing four feet into each square scored 1 horizontal point, and standing up-right on two rear feet scored 1 vertical point. Total score in the open field test was the sum of horizontal points and vertical points.

All procedures performed in this study were approved by the Ethics Committee of Jining Medical University.

### Samples

After behavioral tests, the mice were fasted for 12 h and anesthetized with 10% chloral hydrate by intraperitoneal injection. Then, blood was extracted from abdominal aorta, and serum was separated. Afterwards, the mice were decapitated, and hippocampal tissues were collected and rinsed with precooled 0.9% physiological saline.

### Quantitative real-time polymerase chain reaction (qRT-PCR)

Tissues (100 mg) were ground into powder in liquid nitrogen, and lysed using 1 mL TRIzol reagent following the manufacturer's manual (Thermo Fisher Scientific, USA). In addition, serum (100 μL) was mixed with 1 mL TRIzol (Thermo Fisher Scientific) for lysis. Total RNA was extracted using the phenol chloroform method (20). The concentration and quality of RNA was measured using ultraviolet spectrophotometry (Nanodrop ND2000, Thermo Fisher Scientific). Then, cDNA was obtained by reverse transcription from 1 μg RNA and stored at -20°C. Reverse transcription of mRNA was performed using TIANScript II cDNA First Strand Synthesis Kit (Tiangen, China), and reverse transcription of miRNA was carried out using miRcute miRNA cDNA First Strand Synthesis Kit (Tiangen).

SuperReal PreMix (SYBR Green) qRT-PCR kit (Tiangen) was used to detect mRNA expression of PAI-1, using β-actin as the internal reference. The sequences of PAI-1 were 5′-TCTCCGCCATCACCAACATT-3′ (forward) and 5′-GAGAGAACTTAGGCAGGATGAGG-3′ (reverse). The sequences of β-actin were 5′-AACCCTAAGGCCAACAGTGAAAAG-3′ (forward) and 5′-TCATGAGGTAGTCTGTGAGGT-3′ (reverse). The reaction system (20 μL) was composed of 10 μL SYBR Premix EXTaq, 0.5 μl upstream primer, 0.5 μL downstream primer, 2 μL cDNA, and 7 μL ddH_2_O. PCR conditions were: initial denaturation at 95°C for 2 min, 40 cycles of denaturation at 95°C for 30 s, annealing at 58°C for 30 s, and elongation at 72°C for 30 s (iQ5; Bio-Rad, USA). The 2^-ΔΔCt^ method (21) was used to calculate the relative expression of PAI-1 mRNA against β-actin. Each sample was tested in triplicate.

The expression of miR-17 was determined by miRcute miRNA RT-PCR Kit (Tiangen), using U6 as the internal reference. The sequences of miR-17 primers were 5′-GCAGGAAAAAAGAGAACATCACC-3′ (forward) and 5′-TGGCTTCCCGAGGCAG-3′ (reverse). The sequences of U6 primers were 5′-CTCGCTTCGGCAGCACA-3′ (forward) and 5′-AACGCTTCACGAATTTGCGT-3′ (reverse). The reaction system (20 μL) contained 10 μL qRT-PCR-Mix, 0.5 μL upstream primer, 0.5 μL downstream universal primer, 2 μL cDNA, and 7 μL ddH_2_O. The reaction protocol was: initial denaturation at 95°C for 5 min, 40 cycles of denaturation at 95°C for 10 s, annealing at 60°C for 20 s, and elongation at 72°C for 10 s (iQ5; Bio-Rad). The 2^-ΔΔCt^ method (21) was used to calculate the relative expression of miR-17 against U6. Each sample was tested in triplicate.

### Western blotting

Before lysis, tissues were ground into powder, and cells were trypsinized and collected. Then, tissue samples or cells were lysed with precooled Radio-Immunoprecipitation Assay (RIPA) lysis buffer (600 μL; 50 mM Tris-base, 1 mM EDTA, 150 mM NaCl, 0.1% sodium dodecyl sulfate, 1% TritonX-100, 1% sodium deoxycholate; Beyotime Institute of Biotechnology, China) for 30 min on ice. The mixture was centrifuged at 10,800 *g* for 10 min at 4°C. The supernatant was used to determine protein concentration by bicinchoninic acid (BCA) protein concentration determination kit (RTP7102, Real-Times Biotechnology Co., Ltd., China). The samples were then mixed with 5× sodium dodecyl sulfate loading buffer before denaturation in boiling water bath for 10 min. Afterwards, the samples (20 µg) were subjected to 10% sodium dodecyl sulfate-polyacrylamide gel electrophoresis at 100 V. The resolved proteins were transferred to polyvinylidene difluoride membranes on ice (100 V, 2 h) and blocked with 5% skimmed milk at room temperature for 1 h. Then, the membranes were incubated with rabbit anti-mouse PAI-1 (1:1000; ab7205; Abcam, UK) or β-actin (1:5000; ab8227; Abcam) monoclonal primary antibodies at 4°C overnight. After extensive washing with phosphate-buffered saline with Tween 20 for 15 min 3 times, the membranes were incubated with goat anti-rabbit horseradish peroxidase-conjugated secondary antibody (1:3,000; ab6721; Abcam) for 1 h at room temperature before washing with phosphate-buffered saline with Tween 20 for 15 min 3 times. Then, the membrane was developed with enhanced chemiluminescence detection kit (ab65623; Abcam) for imaging. Image lab v3.0 software (Bio-Rad) was used to acquire and analyze imaging signals. The relative contents of target proteins are reported against β-actin.

### Enzyme-linked immunosorbent assay (ELISA)

PAI-1 ELISA kit (ab157529; Abcam) was used to determine the concentration of PAI-1. On microplates, standards (50 μL) and samples (10 μL serum and 40 μL diluent) were added into predefined wells, while blank wells were left empty. In the wells for standards and samples, horseradish peroxidase-labelled conjugates (100 μL) were added before sealing the plates for incubation at 37°C for 1 h. After washing the plates 5 times, substrates A (50 μL) and B (50 μL) were added to each well. After incubation at 37°C for 15 min, stop solution (50 μL) was added to each well, and absorbance was measured at 450 nm within 15 min.

### Bioinformatics

**Figure 1:**
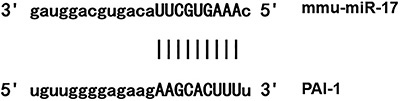
Direct interaction between microRNA-17 and plasminogen activator inhibitor-1 (PAI-1).

Bioinformatics prediction is a powerful tool for the study of the functions of miRNAs. To understand the regulatory mechanism of PAI-1, we used miRanda (http://www.microrna.org/microrna/home.do), TargetScan (http://www.targetscan.org), PiTa (http://genie.weizmann.ac.il/pubs/mir07/mir07_data.html), RNAhybrid (http://bibiserv.techfak.uni-bielefeld.de/rnahybrid/), and PICTA (http://pictar.mdc-berlin.de/) to predict miRNA molecules that might regulate PAI-1, and found that miR-17 was potentially able to regulate PAI-1 (Figure 1).

### Dual luciferase reporter assay

According to the bioinformatics results, wild-type (WT) and mutant seed regions of miR-17 in the 3′-UTR of PAI-1 gene were chemically synthesized *in vitro*. Their two ends were attached with *Spe-1* and *HindIII* restriction sites, and then cloned into pMIR-REPORT luciferase reporter plasmids. Plasmids (0.8 μg) with WT or mutant 3′-UTR sequences were co-transfected with agomiR-negative control (NC) or agomiR-17 (100 nM; Sangon Biotech, China) into 293T cells. After cultivation for 24 h, the cells were lysed using dual luciferase reporter assay kit (Promega, USA) according to the manufacturer's manual, and luminescence intensity was measured using GloMax 20/20 luminometer (Promega). Using renilla luminescence activity as the internal reference, luminescence values of each group of cells were measured.

### Statistical analysis

The results were analyzed using SPSS 20.0 statistical software (IBM, USA), and the data are reported as means±SD. Data were tested for normality. Multigroup measurement data were analyzed using one-way ANOVA. In case of homogeneity of variance, least significant difference and Student-Newman-Keuls methods were used; in case of heterogeneity of variance, Tamhane's T2 or Dunnett's T3 method was used. Comparison between two groups was carried out using Student's *t*-test. P<0.05 indicated statistically significant differences.

## Results

### Mouse depression model was successfully constructed

To confirm the successful construction of the mouse depression model, body weight, sucrose water consumption, and open field activity were examined. The body weight of mice of depression group was not significantly different from that of control group before stimulation (P>0.05), while that of depression group was significantly lower than that of control group on days 28 and 35 after the beginning of stimulation (P<0.05). Before stimulations, the percentage of sucrose water consumption was not different between the two groups (P>0.05), but the percentage of sucrose water consumption in the depression group was significantly lower than that of the control group on days 28 and 35 after the beginning of stimulation (P<0.05). The open field test showed that the total open field score was not significantly different between groups before stimulation (P>0.05), but the total open field score of the depression group was significantly lower than that of the control group (P<0.05) (Table 1). These results suggested that mice in the depression group had depression symptoms and the depression model was successfully constructed.

**Table 1:** Successful construction of mouse depression model.

	Control group	Depression group
Body weight		
Before stimulation	20.51±6.02	19.29±7.23
Day 28	37.28±8.53	31.56±9.32**
Day 35	41.79±9.85	33.23±8.81**
Sucrose water consumption (%)		
Before stimulation	93.6±4.6	95.1±3.8
Day 28	94.1±5.3	86.8±8.2*
Day 35	91.2±7.3	77. 9±7.8*
Total score of open field test		
Before stimulation	125.6±32.3	128.1±26.8
Day 28	128.6±30.3	72.8±21.9**
Day 35	116.9±22.1	56.2±16.8**

Data are reported as means±SD for n=20. *P<0.05, **P<0.01, compared with the control group (*t*-test).

### Mice with depression had elevated levels of PAI-1 mRNA in hippocampal tissues and blood

**Figure 2:**
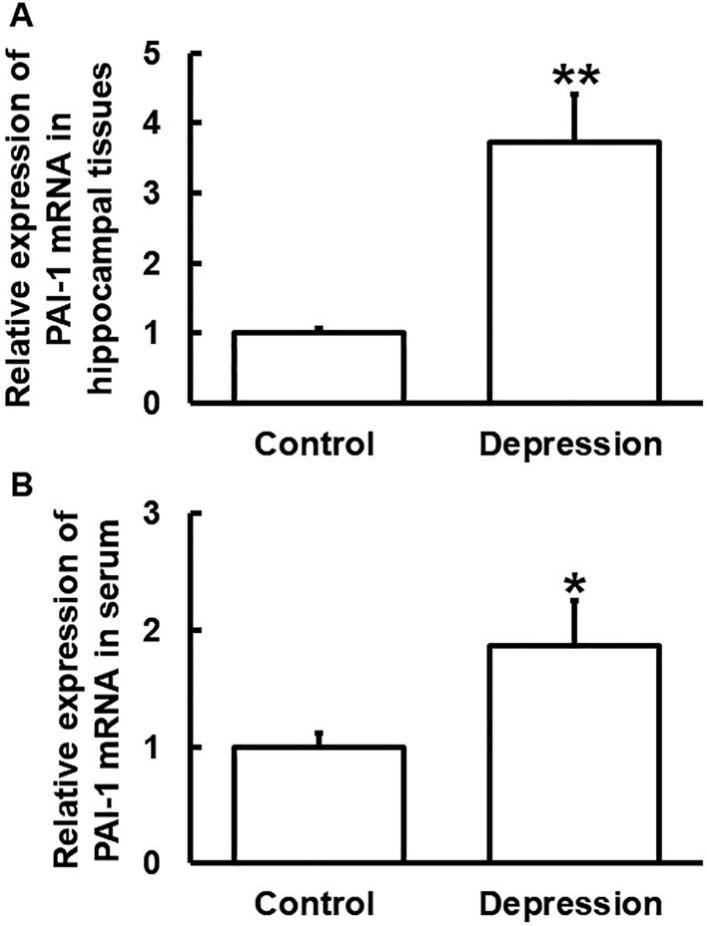
Relative expression of plasminogen activator inhibitor-1 (PAI-1) mRNA in (**A**) hippocampal tissues and (**B**) serum from mice in the control and depression groups. Expression of mRNA was determined by qRT-PCR. Data are reported as means±SD. *P<0.05 and **P<0.01 compared with control (*t*-test).

The levels of PAI-1 mRNA in hippocampal tissues and serum from mice in the depression group were significantly higher than those in the control group (P<0.05) (Figure 2A and B).

### Expression of PAI-1 protein in hippocampal tissues and blood from mice with depression was up-regulated

**Figure 3:**
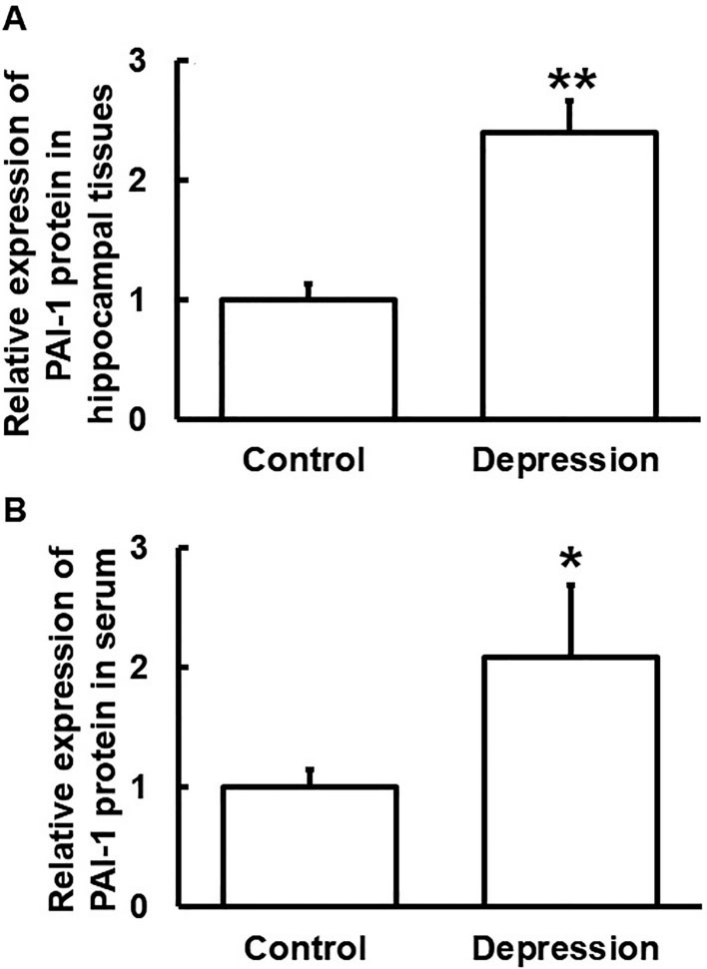
Relative expression of plasminogen activator inhibitor-1 (PAI-1) protein in (**A**) hippocampal tissues and (**B**) serum from mice in the control and depression groups. Expression of protein in hippocampal tissues and serum were determined by Western blotting and ELISA, respectively. Data are reported as means±SD. *P<0.05 and **P<0.01 compared with control (*t*-test).

PAI-1 protein expression in hippocampal tissues and serum from mice in the depression group was significantly higher than that in the control group (P<0.05) (Figure 3A and B), being consistent with that of PAI-1 mRNA.

### Expression of miR-17 was decreased in hippocampal tissues and blood from the depression model mice

**Figure 4:**
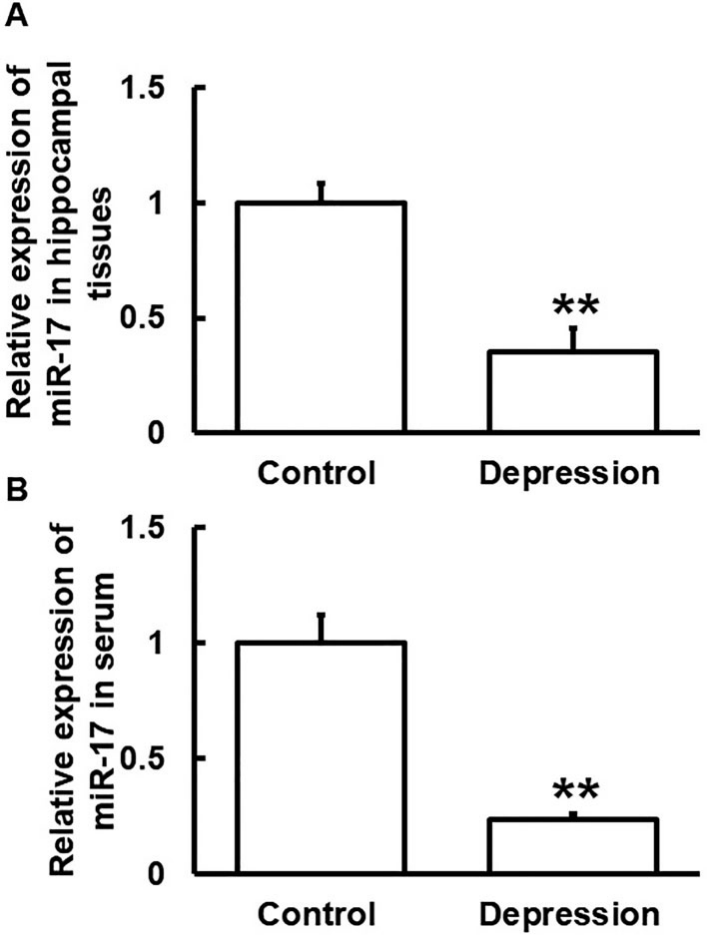
Relative expression of miR-17 in (**A**) hippocampal tissues and (**B**) serum from mice in the control and depression groups. Expression of miR-17 was determined by qRT-PCR. Data are reported as means±SD. **P<0.01 compared with control (*t*-test).

The expression of miR-17 in hippocampal tissues and serum from mice in the depression group was significantly lower than that in the control group (P<0.05) (Figure 4).

### miR-17 could bind with the 3′-UTR seed region of PAI-1 mRNA to regulate its expression

**Figure 5:**
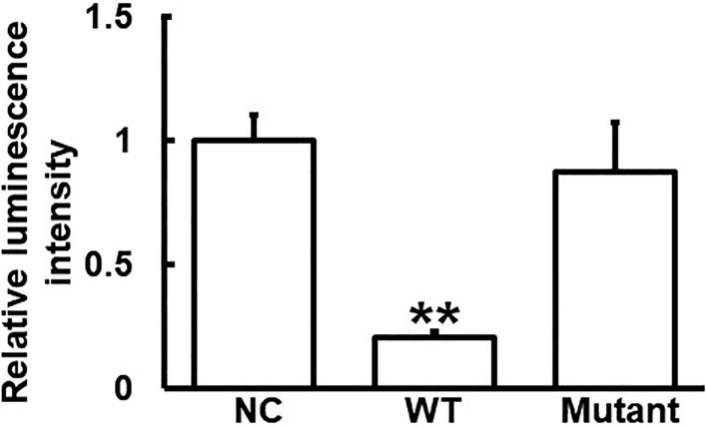
Identification of interaction between miR-17 and plasminogen activator inhibitor-1 (PAI-1) mRNA using dual luciferase reporter assay. Data are reported as means±SD. **P<0.01 wild type (WT) compared with negative control (NC) (ANOVA).

The fluorescence value of cells co-transfected with agomiR-17 and pMIR-REPORT-WT PAI-1 luciferase reporter plasmids was significantly lower than that of the negative control group (P<0.05). By contrast, the fluorescence value of cells co-transfected with agomiR-17 and pMIR-REPORT-mutant PAI-1 luciferase reporter plasmids was not significantly different from that of the negative control group (P>0.05) (Figure 5). These results indicated that miR-17 could bind with the 3′-UTR seed region of PAI-1 mRNA to regulate its expression.

## Discussion

The theoretical basis of CUMS animal model is similar to the mechanism underlying the occurrence and development of depression caused by chronic and low-level stressors in humans. Using this model, stress-induced behavioral abnormality can be maintained for several months, and antidepressants can correct these abnormal behaviors (17). Therefore, the model has become one of the most widely used models of depression (22).

One of the pathological features of the brain is amyloid lesions of cortical artery and arterioles. It is reported that PAI-1 has a close relationship with vascular lesions. PAI-1 not only affects thrombosis, but also participates in the accumulation of extracellular matrix and migration of smooth muscle cells, inducing the combination of low-density lipoprotein with vascular smooth muscle cells (23). PAI-1 is deposited in extracellular matrix, promotes the formation of lipoid and atheromatous plaque, thickens basement membranes, and hardens vessel walls, thus promoting the occurrence and development of vascular diseases (24). PAI-1 participates in the hydrolysis of brain-derived neurotrophic factor (BDNF) to regulate the growth and apoptosis of neurons, which are thought to be associated with depression (25). In addition, the tPA/PAI-1-plasmin system that involves PAI-1 can inhibit depressive behaviors induced by excitable neurotoxicity, which is caused by excessive activation of N-methyl-D-aspartic acid receptor (NMDA) (26). In our mouse model of depression, PAI-1 mRNA and protein expression were up-regulated in both hippocampal tissues and blood, suggesting that PAI-1 up-regulation may be a key factor in the pathological process of depression in mice.

Regulating the transcription and expression of mRNA is a complex process with multiple factors. miRNA molecules can achieve negative feedback regulation on their target mRNA by cutting the mRNA and inhibiting its translation (27). miRNA is an important regulator in normal development, physiology, and diseases, and many miRNA molecules have become biomarkers of various diseases (28). We used bioinformatics to predict the upstream genes that regulate PAI-1 and referred to relevant literature on miRNA that can regulate PAI-1. We found that miR-17 may be one of the upstream target genes regulating PAI-1. In cells expressing proto oncogene c-Myc, the expression level of miR-17 is up-regulated, and the expression level of transcription regulator E2F is reduced, suggesting that miR-17 has the effect of promoting cell proliferation mediated by c-Myc (29). In the field of cancer research, miR-17 has been proven to have functions of promoting tumor cell proliferation and inhibiting tumor cell apoptosis (30,31).

In the present study, expression of miR-17 was down-regulated, in contrast to the up-regulation of PAI-1 mRNA and protein in depressed mice. This suggested that the occurrence of depression may be related to the down-regulation of miR-17. Our results of dual luciferase reporter assay showed that miR-17 directly binds to the 3′-UTR seeding region of PAI-1 mRNA and regulates the expression of PAI-1 mRNA. These results indicated that miR-17, PAI-1, and depression have a regulatory relationship. Changes of miR-17 levels in blood may be a diagnostic marker for depression. However, deeper studies into the mechanism by which miR-17 regulates PAI-1 are still needed in the future.

In conclusion, the present study demonstrated that decreased expression of miR-17 in hippocampal tissues and blood from mice with depression up-regulated the expression of PAI-1 mRNA and protein. As a strong regulatory factor of PAI-1, miR-17 may become a new research hot spot for the prevention and treatment of depression.
